# Screening, validation, and mechanism study of antitumor components from *Dioscorea nipponica* Makino subsp. *rosthornii* based on spectrum-effect relationship analysis

**DOI:** 10.3389/fonc.2026.1836431

**Published:** 2026-04-29

**Authors:** Mengjia Liang, Moyu Shen, Yuxuan Wang, Pengxin Fan, Yuguang Zheng, Haiyang Wang, Jingcun Sun, Songsong Jing

**Affiliations:** 1 Traditional Chinese Medicine Processing Technology Innovation Centre of Hebei Province, College of Pharmacy, Hebei University of Chinese Medicine, Shijiazhuang, China; 2 International Joint Research Center on Resource Utilization and Quality Evaluation of Traditional Chinese Medicine of Hebei Province, School of Pharmacy, Hebei University of Chinese Medicine, Shijiazhuang, China; 3 Department of Pharmaceutical Engineering, Hebei Chemical and Pharmaceutical College, Shijiazhuang, China; 4 Department of pharmacy, Cangzhou Hospital of Integrated Traditional Chinese and Western Medicine, Hebei, Cangzhou, China

**Keywords:** A549 cells, antitumor effect, *Dioscorea nipponica* Makino subsp. rosthornii, spectrum-effect relationship, UPLC-QTOF-MS

## Abstract

**Objectives:**

This study aimed to screen potential antitumor compounds from *Dioscorea nipponica* Makino subsp. *rosthornii*, validate them in cellular models, and investigate their mechanisms underlying the inhibition of proliferation in A549 cells.

**Methods:**

The chemical constituents in 12 batches of *D. nipponica* Makino subsp. *rosthornii* were identified using ultra-performance liquid chromatography coupled with quadrupole time-of-flight mass spectrometry (UPLC-QTOF-MS). The antitumor activity of these samples was assessed in A549 cells through cell proliferation, wound healing, migration, and invasion assays. A multivariate regression spectrum-effect relationship model was developed, treating the peak areas of common chromatographic peaks as independent variables and the antitumor activity as the dependent variable. Potential antitumor components were identified based on this spectrum-effect correlation and subsequently validated. The protein expression levels of N-cadherin, MMP2, and E-cadherin in A549 cells were measured to investigate the underlying mechanisms.

**Results:**

A comprehensive chemical analysis was conducted on 12 batches of *D. nipponica* Makino subsp. *rosthornii* from varied geographical origins using UPLC-QTOF-MS, resulting in the identification of 23 distinct chemical constituents. The extract derived from *D. nipponica* Makino subsp. *rosthornii* notably inhibited the proliferation, migration, and invasion of A549 cells. Spectrum-effect relationship analysis pinpointed three potential antitumor compounds: protodioscin, diosgenin, and gracillin. Further mechanistic investigations demonstrated that these saponins inhibit the malignant phenotype of A549 cells through a dual regulatory mechanism. Specifically, they markedly reduced the expression of pro-metastatic proteins N-cadherin and MMP2, while simultaneously enhancing the expression of E-cadherin.

**Conclusion:**

This study elucidated the key active constituents responsible for the antitumor effects of *D*. *nipponica* Makino subsp. *rosthornii*, thereby providing scientific evidence to support its potential application in cancer therapy.

## 1 Introduction

Lung cancer is among the most prevalent malignant tumors globally and is the leading cause of cancer-related mortality (1). In recent years, the increasing incidence and mortality of cancer have made the research and development of anti-cancer drugs a critical concern and a global priority in scientific research. Traditional Chinese medicine (TCM) is a promising source of novel anti-cancer agents, owing to its well-established clinical history, low toxicity, and multifaceted mechanisms of action (2). A hallmark of cancer cells is their capacity for uncontrolled proliferation and metastasis to distant tissues (3, 4). The migration, invasion, and adhesion of tumor cells are crucial processes that drive tumor progression and metastatic dissemination (5–7).

Plants of *Dioscorea* genus (Dioscoreaceae) produce a diverse array of active ingredients, including saponins, flavonoids, steroids, phenols and diaromatic heptane (8–10). Modern pharmacological studies have shown that *Dioscorea* plants have a variety of pharmacological effects, including hypoglycemic (11), anti-inflammatory (12), antitumor (13) and analgesic. *Dioscorea nipponica* Makino subsp. *rosthornii* (Prain et Burkill) C. T. Ting is a rhizomatous species of the genus *Dioscorea* (family Dioscoreaceae) (8, 9). Taxonomically, it is classified as a subspecies of *D. nipponica* Makino. The nominate subspecies, *D. nipponica* Makino, has been demonstrated to possess antitumor activity, and its chemical constituents are predominantly steroidal saponins, which are considered the main active components responsible for the antitumor effects of *Dioscorea* plants. Both *D. nipponica* Makino subsp. *rosthornii* and *D. nipponica* Makino share steroidal saponins as their major chemical constituents, with most of these saponins abundantly present in both subspecies. However, some compounds have been reported exclusively in *D. nipponica* Makino subsp. *rosthornii*, while they have not been found in *D. nipponica* Makino (9), indicating certain chemical differences between the two closely related subspecies. Given the well−established antitumor activity of *D. nipponica* Makino and the unique chemical profile of *D. nipponica* Makino subsp. *rosthornii*, investigating the antitumor effects of the latter is of great significance.

The efficacy of herbal medicines depends on the synergistic interaction of multiple components. Consequently, the identification of active compounds via conventional methods—extraction, purification, structural characterization, and bioactivity screening—remains a laborious and often challenging endeavor. Spectral potency analysis is a reliable technique for correlating the bioactivity of herbs through chemical fingerprinting, thereby enabling the identification of biologically active ingredients (14). Spectral effect relationship analysis, an approach that integrates chemical fingerprint data with pharmacodynamic assessments, directly correlates specific spectral features with their corresponding bioactivities. Therefore, spectrum-effect relationship analysis serves as a widely used tool for evaluating and screening active ingredients in traditional Chinese medicine.

Therefore, this study first characterized the chemical composition of *D. nipponica* Makino subsp. *rosthornii* using Ultra-Performance Liquid Chromatography Coupled to Quadrupole Time-of-Flight Mass Spectrometry (UPLC-QTOF-MS). Subsequently, through *in vitro* experiments with A549 cells integrated with spectrum-effect relationship analysis, potential antitumor constituents were screened and validated. Furthermore, the mechanism responsible for inhibiting A549 cell proliferation was elucidated.

## 2 Materials and methods

### 2.1 Materials and reagents

12 batches of *D. nipponica* Makino subsp. *rosthornii* samples were obtained as commercially available materials from different regions in China. The sources of the samples are listed in **Table 1**. All samples were collected in December, and the processing method was uniformly applied as follows: removing sediment, cutting into segments, and natural drying. The voucher specimens were identified by Associate Professor Songsong Jing and have been deposited at the Traditional Chinese Medicine Processing Technology Innovation Center of Hebei Province, Hebei University of Chinese Medicine.

**Table 1:** Sample information.

Sample number	Origins	Sample number	Origins
S1	Guizhou province, China	S7	Sichuan province, China
S2	Guizhou province, China	S8	Hubei province, China
S3	Guizhou province, China	S9	Hubei province, China
S4	Guizhou province, China	S10	Shaanxi province, China
S5	Sichuan province, China	S11	Shaanxi province, China
S6	Sichuan province, China	S12	Chongqing, China

Reference standards of dioscin, gracillin, protodioscin and rutin were purchased from Chengdu PUSH Bio-Technology Co., Ltd. (Chengdu, China). HPLC-grade methanol, acetonitrile, and formic acid were purchased from Fisher Scientific (Pittsburgh, PA, USA). Ultrapure water was prepared using a Synergy water purification system (Millipore, Billerica, USA). All other chemicals and reagents were of analytical grade.

The human non-small cell lung cancer A549 cells were purchased from Wuhan Shangen Biotechnology Co. (Wuhan, China). Fetal bovine serum (FBS) and Roswell Park Memorial Institute 1640 medium (RPMI 1640) were purchased from GIBCO (New York, USA). Penicillin-streptomycin solution, trypsin, and the Cell Counting Kit-8 (CCK-8) were purchased from Solarbio (Beijing, China). Transwell chambers (8 µm pore size) were purchased from Beijing Lanjieke Technology Co. (China), and the Matrigel^®^ matrix was purchased from Corning Corporation (USA). Antibodies against E-cadherin, N-cadherin, matrix metalloproteinase 2 (MMP2), Kelch-like ECH-associated protein 1 (Keap1), nuclear factor erythroid 2-related factor 2 (Nrf2), heme oxygenase 1 (HO-1), and *β*-actin were purchased from Affinity Biosciences.

### 2.2 UPLC-QTOF-MS analysis

#### 2.2.1 Samples pretreatment

The *D. nipponica* Makino subsp. *rosthornii* samples were powdered and screened through 60 mesh sieves. 1.0 g of sample powder was extracted with 20 mL of 70% ethanol by sonication for 20 min, followed by centrifugation at 13,000 rpm for 10 min. The supernatants were diluted 80-fold with extraction solution, filtered through a 0.22 μm pore size filter, and transferred to sample vials. Then, the internal standard (rutin) was added to each analytical sample to achieve a final concentration of approximately 0.02 mg/mL. The prepared samples are then subjected to UPLC−QTOF−MS analysis. Quality control (QC) samples were prepared by pooling sample extracts and were used to assess the repeatability of the method for samples processed under identical conditions. A QC sample was inserted after every five test and analysis samples.

#### 2.2.2 Chromatographic conditions

The mobile phases employed were a 0.1% formic acid aqueous solution (A) and acetonitrile (B), and the gradient elution conditions were as follows: The mobile phases were as follows: 0–5 min, 10%-20% B; 5–12 min, 20%-30% B; 12–16 min, 30%-35% B; 16–22 min, 35%-40% B; 22–26 min, 40%-50% B; 26–34 min, 50%-83% B. The injection volume was 10 μL and the flow rate was 1 mL/min.

#### 2.2.3 Mass spectrometry conditions

The parameters employed for the MS acquisition were as follows: capillary voltage, 3500 V; fragmentation voltage, 135 V; dry gas (N_2_) temperature, 320 °C; sheath gas temperature, 350 °C; gas flow rate, 10 L/min; and sheath gas flow rate, 11 L/min. Nebulizer gas pressure was set at 35 psi, and the collision energies were set to 20 eV and 40 eV. The mass scan was conducted in the range m/z 100-1000, and the analysis was performed in positive ion mode. The data were acquired on a Mass Hunter workstation (Agilent Technologies Co., Ltd., USA).

#### 2.2.4 Data processing and analysis

Qualitative analysis of the chemical components in *D. nipponica* Makino subsp. *rosthornii* was performed through literature retrieval and searches of the PubChem and MassBank databases. Using the Qualitative Navigator (B.08.00) software, the measured total ion current chromatogram of *D. nipponica* subsp. *rosthornii* was compared, along with the accurate precursor and fragment masses of the compounds, to identify each compound. For quantitative analysis, the peak areas of the corresponding peaks in each sample were extracted using the Quant Analysis (B.09.00) batch processing software. Normalization and relative quantification of each compound were carried out; the integrated peak area was used as a variable for analysis and normalized to the internal standard. The UPLC−QTOF−MS dataset was then transformed to a range of -1 to 1 using the Min−Max normalization method.

### 2.3 Antitumor activity of *D. nipponica* Makino subsp. *rosthornii* samples

#### 2.3.1 Cell cultivation

A549 cells were maintained in RPMI 1640 medium supplemented with 10% FBS and 1% penicillin-streptomycin at 37°C in a 5% CO_2_ humidified atmosphere. Upon reaching 80% confluence, the cells were subcultured using 0.25% trypsin.

#### 2.3.2 Proliferation assay using CCK-8

A549 cells in the logarithmic growth phase were seeded into 96-well plates at a density of 7×10³ cells per well. After incubation under standard conditions until 70–80% confluence, the medium was replaced with fresh RPMI-1640 containing *D. nipponica* Makino subsp. *rosthornii* ethanol extract. Final extract concentrations were 250, 300, 350, 400, and 450 µg/mL, with six replicate wells per concentration. Following treatment for 12, 24, 36, or 48 h, CCK-8 reagent was added and the plates were incubated for another hour. Absorbance was then measured at 450 nm. Cell viability was calculated as: [(OD experimental – OD blank)/(OD control – OD blank)] ×100%. The half-maximal inhibitory concentration (IC_50_) was derived from the resulting data.

#### 2.3.3 Wound healing experiment

Initially, the bottoms of 6-well plates were marked with horizontal lines at 0.5–1 cm intervals, ensuring at least five lines were marked per well. A549 cells were then seeded at a density of 5×10_5_ cells per well and incubated overnight to form a confluent monolayer. On the following day, a sterile pipette tip was used to create scratch wounds perpendicular to the pre-drawn lines. Detached cells were removed by washing the wells three times with PBS, after which fresh serum-free medium was added to visualize the scratch wounds. The cells were incubated at 37°C with 5% CO_2_, and images of the same fields were captured at 0, 24, and 48 h post-scratching. The migration area was quantified by measuring the scratch closure using ImageJ software.

#### 2.3.4 Cell migration assay

Upon reaching 90% confluence, cells were washed with PBS and detached using trypsin. Digestion was monitored under a microscope and terminated when cells rounded up. The cell suspension was centrifuged at 900 × g for 3 min, and the pellet was washed twice with PBS. Cells were then resuspended in serum-free RPMI-1640 medium and adjusted to a density of 1 × 10_5_ cells/mL. For the migration assay, 500 μL of complete medium was added to the lower chamber of a Transwell plate. A 200 μL aliquot of the cell suspension was carefully added to the upper chamber, avoiding bubble formation. After 24 h of incubation at 37°C, the cells that had migrated to the lower side of the membrane were fixed with 4% paraformaldehyde for 25 min, stained with 0.1% crystal violet for 30 min, and gently wiped with a cotton swab to remove non-migrated cells. The migrated cells were counted in 3 random fields per membrane under an inverted microscope.

#### 2.3.5 Cell invasion assay

Matrigel was aliquoted into 100 μL volumes in pre-cooled microtubes on ice and stored at -20°C. All tools, including serum-free RPMI-1640 medium, pipette tips (100 μL and 1000 μL), and the Transwell chambers, were pre-cooled to 4°C to prevent premature gelation. After defrosting the Matrigel, the Matrigel dilution was prepared at a ratio of 1640 medium (without FBS) to Matrigel of 1:37 and swirled gently to mix. Slowly add 100 µL of the Matrigel dilution along the inner wall of the cell insert. The cell inserts were placed in 24-well plates and incubated at 37°C for 1 h to allow the Matrigel dilution to solidify as a matrix gel. Subsequent experimental steps followed the procedure described in Section 2.3.4.

### 2.4 Relationship analysis of spectral effects

#### 2.4.1 Pearson correlation analysis

Pearson’s correlation coefficient is a statistical method that is widely used in the analysis of the spectral correlation of active ingredients in traditional Chinese medicine (15). The peak areas of the 23 common peaks of *D. nipponica* Makino subsp. *rosthornii* samples in positive ion mode were selected as the independent variables, while the antitumor activity indicators—cell wound healing rate, migration rate, and invasion rate were selected as dependent variables. Pearson correlation analysis was introduced into SPSS 23.0 software to calculate the Pearson correlation coefficients between the cell mobility values and the peak areas of the common peaks; the larger the absolute value of the correlation coefficients, the stronger the correlation, and the compounds with strong antitumor activity were selected as the screening results.

#### 2.4.2 Regression analysis (partial least squares)

Partial Least Squares Regression (PLS regression) is a technique that combines multivariate statistical methods with Principal Component Analysis (PCA) to effectively deal with a limited number of samples and a large number of independent variables by constructing a highly accurate data model. The method uses regression coefficients as evaluation criteria to visually show the relative influence of each predictor variable on the outcome variable. The normalized peak areas of the 23 shared peaks were used as the independent variables (X), and cell migration was used as the dependent variable (Y), and SIMCA 14.0 was used to determine the regression coefficients and calculate the VIP values. When the VIP value was > 1, the compound represented by the shared peak was considered to have significant antitumor effect.

### 2.5 Validation of antitumor activity

To further validate the antitumor activity of potential antitumor compounds in A549 cells, CCK-8 cell proliferation assay, wound healing assay, cell invasion assay, and migration assay were performed according to the methods described in Section 2.3.

### 2.6 Potential mechanism of the antitumor activity of *D. nipponica* Makino subsp. *rosthornii*

#### 2.6.1 Western blot analysis

After harvesting, the cells were lysed with pre-cooled RIPA buffer. Following protein quantification, the protein samples were denatured by heating at 95°C for 10 min in a metal bath. The proteins were separated using 10% SDS-PAGE gels and subsequently transferred to polyvinylidene fluoride (PVDF) membranes via electrophoresis. The membranes were blocked with 5% BSA-TBST and incubated with primary antibodies overnight at 4°C. The next day, the membranes were incubated with secondary antibodies. Finally, the membranes were thoroughly incubated with enhanced chemiluminescence (ECL) reagent, and the protein bands were visualized using a gel imaging system.

#### 2.6.2 Quantitative real-time PCR

Total mRNA was extracted using a commercial kit and reverse transcribed into cDNA with reverse transcriptase. Quantitative real-time PCR analysis was performed on a real-time PCR system. The primers used for quantitative real-time PCR in this study are listed in **Table 2**, with *β*-actin serving as the internal reference.

**Table 2:** Sample information.

Genes	Sequences (5’-3’)
*β*-actin	S CACCCAGCACAATGAAGATCAAGAT A CCAGTTTTTAAATCCTGAGTCAAGC
MMP2	S AGTGGATGATGCCTTTGCTCG A CAAGGTCCATAGCTCATCGTCAT
E-cadherin	S GAGGATTTTGAGCACGTGAAGAA A GGGAGATGTATTGGGAGGAAGGT
N-cadherin	S AAGAGGCAGAGACTTGCGAAAC A TGGAGTCACACTGGCAAACCTT
HO-1	S ATGCCCCAGGATTTGTCAGAG A GGAAGTAGACAGGGGCGAAGAC
Nrf 2	S TGAGGTTTCTTCGGCTACGTT A CTTCTGTCAGTTTGGCTTCTGG
Keap 1	S CGCCAACTTCGCTGAGCA A GAAGTTCGGCGTCAACGAGT

### 2.7 Statistical analysis

Data are expressed as mean ± SD and were obtained from at least three independent replicates. Statistical significance between two groups was determined using an unpaired Student’s t-test. For multiple comparisons, one-way ANOVA with Tukey’s multiple comparisons test was applied. 95% confidence intervals (CIs) were calculated for the primary outcome measures. All statistical analyses were performed with GraphPad Prism version 8.0. Differences were considered statistically significant at *P* < 0.05.

## 3 Results

### 3.1 UPLC-Q-TOF/MS characterization of common peaks in *D. nipponica* Makino subsp. *rosthornii*

**Figure 1:**
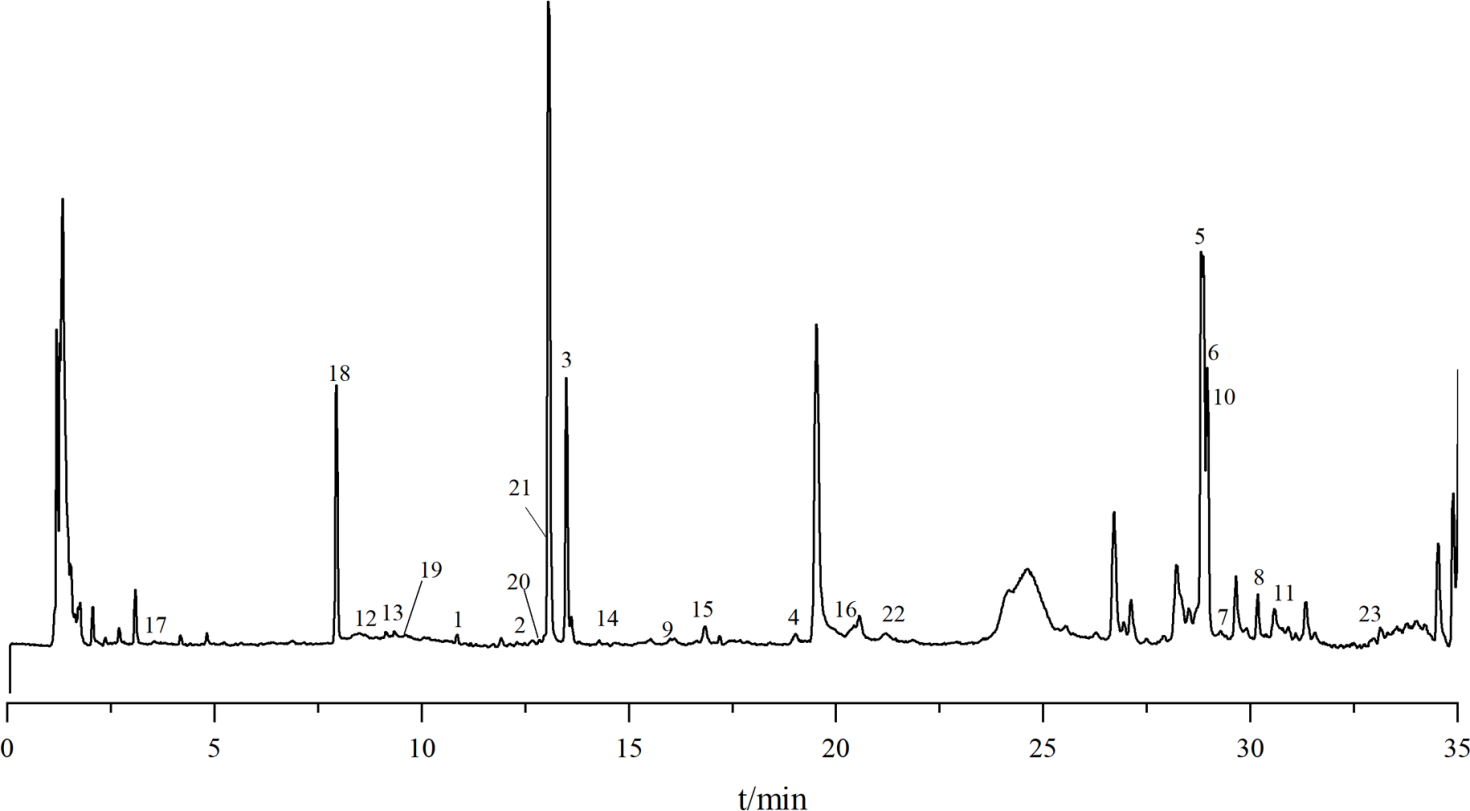
The typical total ion chromatograms of *D. nipponica* Makino subsp. *rosthornii* samples in positive ion mode by UPLC-QTOF-MS.

The sample of *D. nipponica* Makino subsp. *rosthornii* was analyzed by UPLC-Q/TOF-MS, and the molecular formula of the compounds were calculated based on the mass-to-charge ratios of the molecular ion peaks of the compounds using electrospray ionization in positive ion mode. The possible chemical structures were identified and deduced from the fragmentation information of the secondary mass spectra, the mass spectral cleavage pattern of the control products and from the relevant literature. A total of 23 compounds were identified. The total ion flow chromatogram of the *D. nipponica* Makino subsp. *rosthornii* sample is shown in **Figure 1**, and the identified information is presented in **Table 3**.

**Table 3:** Identification of compounds in *D. nipponica* Makino subsp. *rosthornii* using UPLC-QTOF-MS.

No.	T_R_ (min)	Formula	[M+H] ^+^ (*m/z*)	Fragmentions (*m/z*)	Error (ppm)	Identification	Classification	References
1	10.914	C_39_H_62_O_13_	739.4258	577.3731	-0.77	Diosgenin diglucoside	Steroidal saponins	(16)
2	13.073	C_51_H_84_O_22_	1049.5419	1031.5419 577.3726	-0.59	Protodioscin	Steroidal saponins	(17)
3	13.489	C_51_H_84_O_23_	1064.4670	1047.5354 1032.5435 885.482 579.3803 415.3201 129.055	2.34	Protogracillin	Steroidal saponins	(18)
4	15.97	C_43_H_72_O_16_	867.4709	655.4821 517.3946	1.76	Cyclopassifloside III	Triterpenoid saponin	Database
5	19.388	C_27_H_42_O_3_	577.3734	415.3211	-0.27	Diosgenin	Steroidal saponins	(18, 19)
6	28.855	C_45_H_72_O_16_	869.4887	397.3097 293.1226 415.3209 869.4887 870.4920	-0.77	Dioscin	Steroidal saponins	(20)
7	28.944	C_45_H_72_O_17_	885.4841	885.4841 723.4295 577.3735 415.3211 397.3101 309.1176	-0.21	Gracillin	Steroidal saponins	(21)
8	28.95	C_43_H_74_O_17_	885.4833	783.4761 612.34957	-1.46	Cyclotricuspidoside C	Triterpenoid saponin	Database
9	29.011	C_33_H_52_O_8_	577.3726	415.3204 397.3098	-1.51	Disogluside	Steroidal saponins	Database
10	30.143	C_39_H_62_O_12_	723.4308	301.286	-1.23	Prosapogenin A	Steroidal saponins	(21)
11	8.651	C_21_H_20_O_12_	465.1031	303.0489	2.92	Isoquercetin	Flavone	Database
12	9.435	C_28_H_36_O_13_	598.2488	417.1549 181.0501	-1.78	Acanthoside B	Cholestanol	Database
13	13.225	C_37_H_64_O_13_	739.4244	714.4346	0.52	Evasterioside E	Phytosterol	Database
14	15.295	C_15_H_10_O_7_	303.0498	397.3097 293.1226 229.1408 210.0431	-0.54	Quercetin	Flavone	(19, 22)
15	16.555	C_16_H_12_O_5_	285.0755	286.079	-1.04	Physcione	Anthraquinones	Database
16	20.087	C_20_H_18_O_9_	403.1031	269.3717 255.2812	-0.76	Frangulin B	Anthraquinones	Database
17	33.165	C_15_H_22_O	219.1741	301.286	-1.13	Turmerone	Sesquiterpenoids	Database
18	12.899	C_19_H_18_O_4_	311.1277	293.1185	-0.27	Przewaquinone A	Diterpenoid quinones	Database
19	20.422	C_17_H_23_O_12_	420.1282	241.3937 197.3759	4.71	10-carboxyloganin	Iridoid glycosides	Database
20	30.099	C_37_H_64_O_12_	723.4697	455.1834 469.2945	1.45	Fuscaside B	Triterpenoid saponin	Database
21	3.785	C_15_H_20_O_4_	265.1427	187.9733 153.0767	-2.86	(+)-Abscisic acid*	Organic acid	Database
22	8.206	C_27_H_44_O_7_	481.3155	445.2953	-0.75	20-hydroxyecdysone	Phytosterol	Database
23	9.174	C_27_H_30_O_15_	595.1652	287.0538	-0.82	Kaempferol-3-O-rutinoside	Flavone	(19)

### 3.2 Inhibition of A549 cell proliferation by *D. nipponica* Makino subsp. *rosthornii*

**Figure 2:**
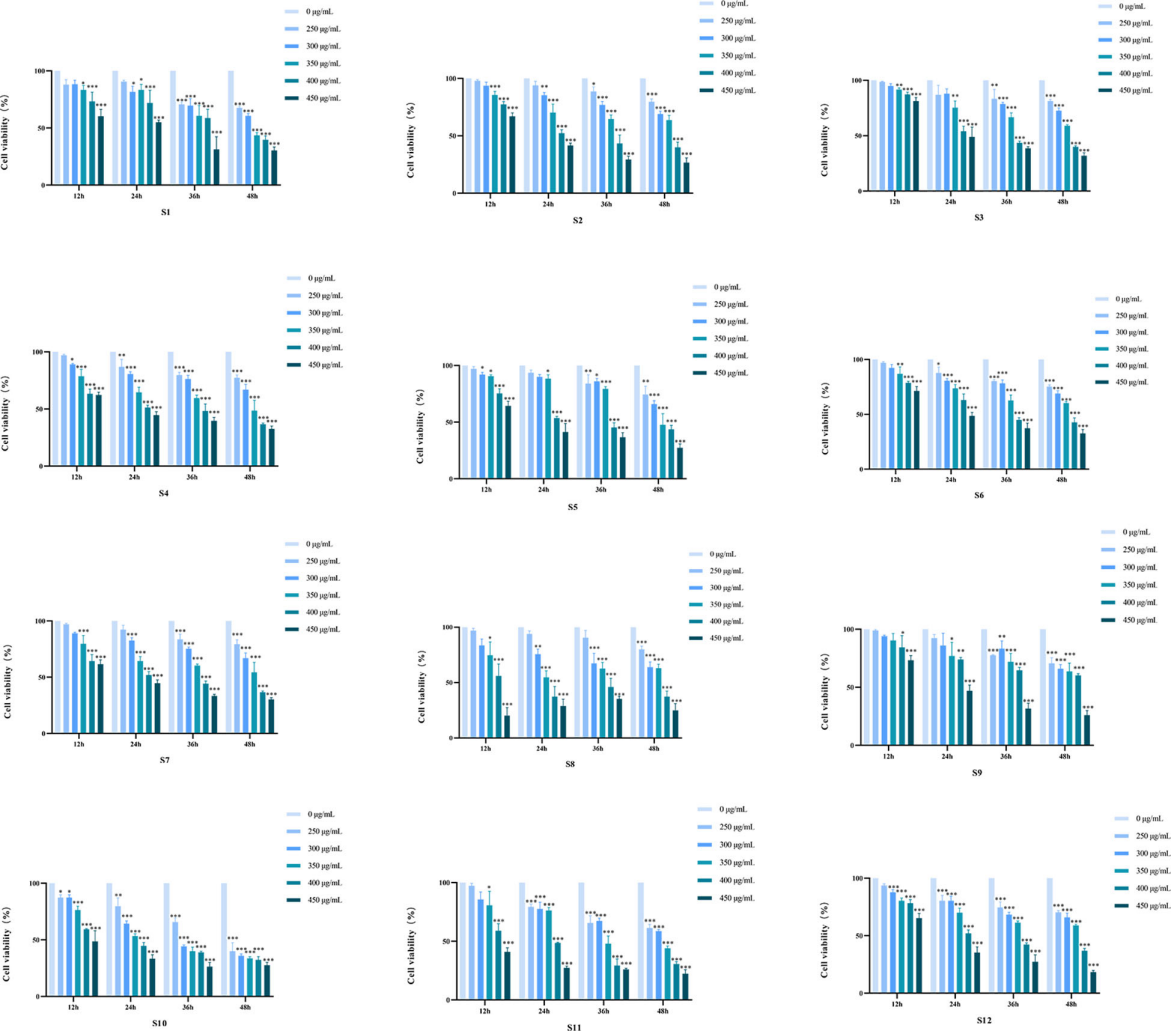
Inhibitory effect of *D. nipponica* Makino subsp. *rosthornii* on A549 cell proliferation (n=3) data are presented as mean ± SD (*P < 0.05, **P < 0.01, ***P < 0.001 vs control group).

The anti-proliferative effect of different batches of *D. nipponica* Makino subsp. *rosthornii* on A549 cells was evaluated using the CCK-8 assay. Cells in the logarithmic growth phase were seeded into 96-well plates and treated with different concentrations of the ethanol extract. After incubation for 0, 12, 24, 36, and 48 h, the CCK-8 solution was added, and the absorbance was measured. As shown in **Figure 2**, the extract inhibited cell proliferation in a concentration- and time-dependent manner relative to the 0-h control. The IC_50_ value for each batch was determined from this data to inform subsequent experiments.

### 3.3 Effect of *D. nipponica* Makino subsp. *rosthornii* on wound healing in A549 cells

**Figure 3:**
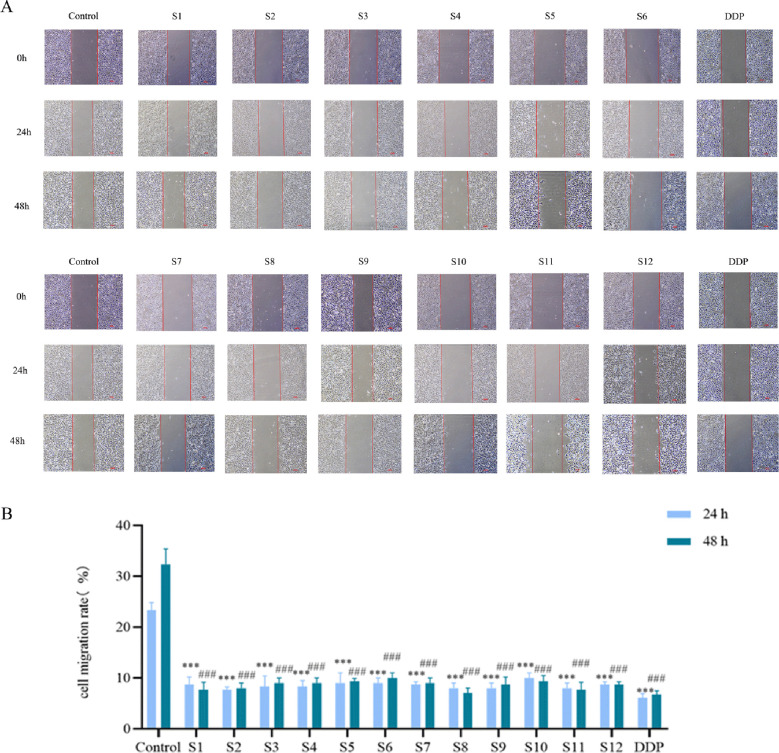
Effect on A549 cell cure rate of *D. nipponica* Makino subsp. *rosthornii* (n=3) **(A)** representative images of wound closure. **(B)** quantitative analysis of wound healing. Data are presented as mean ± SD (*P < 0.05, **P < 0.01, ***P < 0.001 vs 24h Control group; ^#^*P* < 0.05, ^##^*P* < 0.01, ^###^P < 0.001 vs 48 h control group).

A confluent cell monolayer was intentionally scratched to create a cell-free gap. The subsequent migration of cells from the edge into this gap to close it models the process of cell migration *in vivo*. As shown in **Figure 3**, after 24 h and 48 h of treatment, the gap closure in the treatment groups was significantly slower than that in the blank control group. This indicated that *D. nipponica* Makino subsp. *rosthornii* inhibits the migration of A549 cells.

### 3.4 Effect of *D. nipponica* Makino subsp. *rosthornii* on A549 cell migration

**Figure 4:**
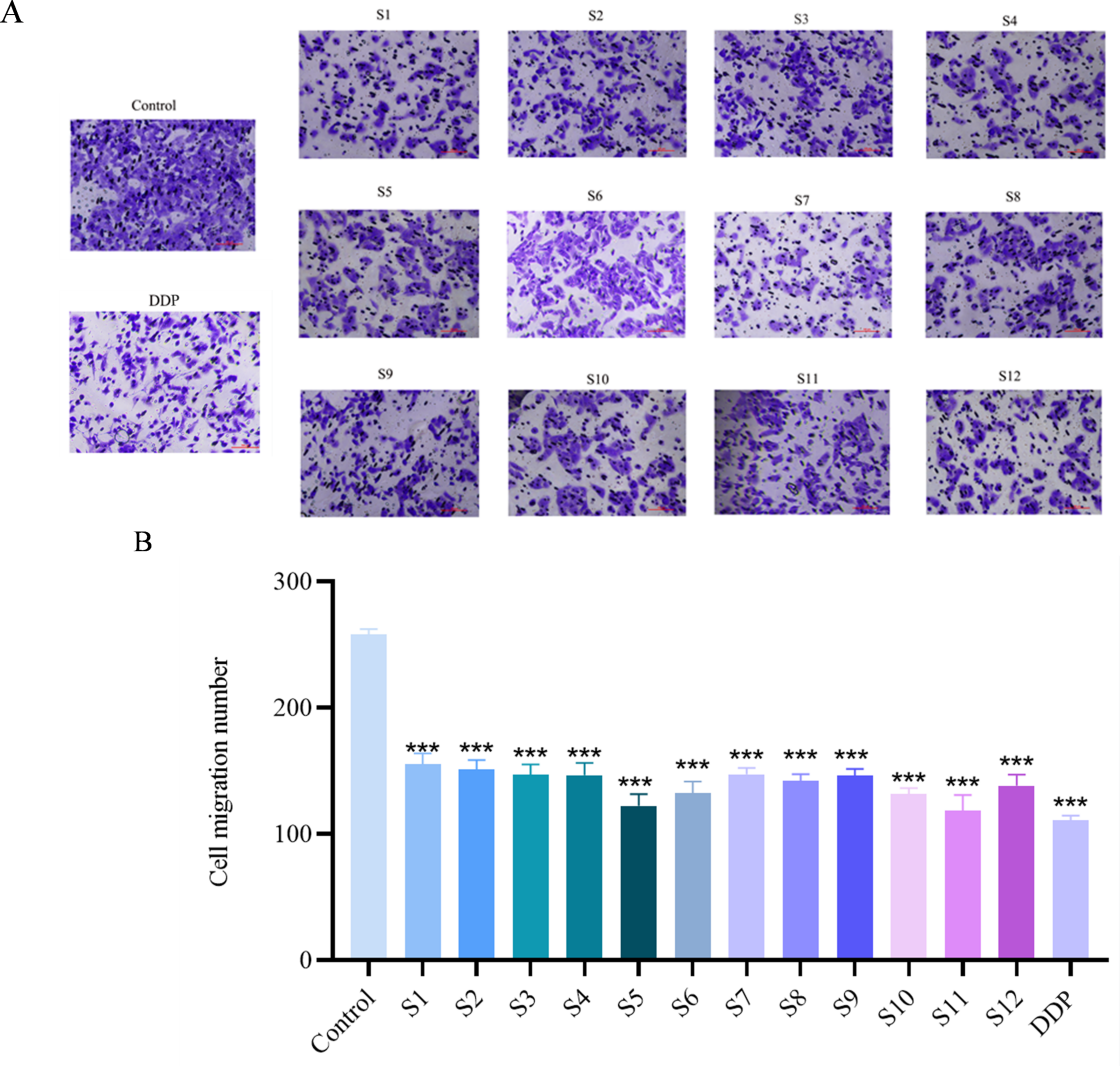
Effect on A549 Cell migration rate of *D. nipponica* Makino subsp. *rosthornii* (n=3) **(A)** Representative microscopic images of cell migration. **(B)** Quantitative analysis of migration rate. Data are presented as mean ± SD (*P < 0.05, **P < 0.01, ***P < 0.001 vs Control group).

Cell migration is essential for multiple stages of cancer progression, including metastasis and angiogenesis (the formation of new blood vessels) (23). As shown in **Figure 4**, the treatment significantly reduced the migration of A549 cells compared to the untreated control, demonstrating the inhibitory effect of *D. nipponica* Makino subsp. *rosthornii*.

### 3.5 Effect of ethanolic extracts of *D. nipponica* Makino subsp. *rosthornii* on A549 cell invasion

**Figure 5:**
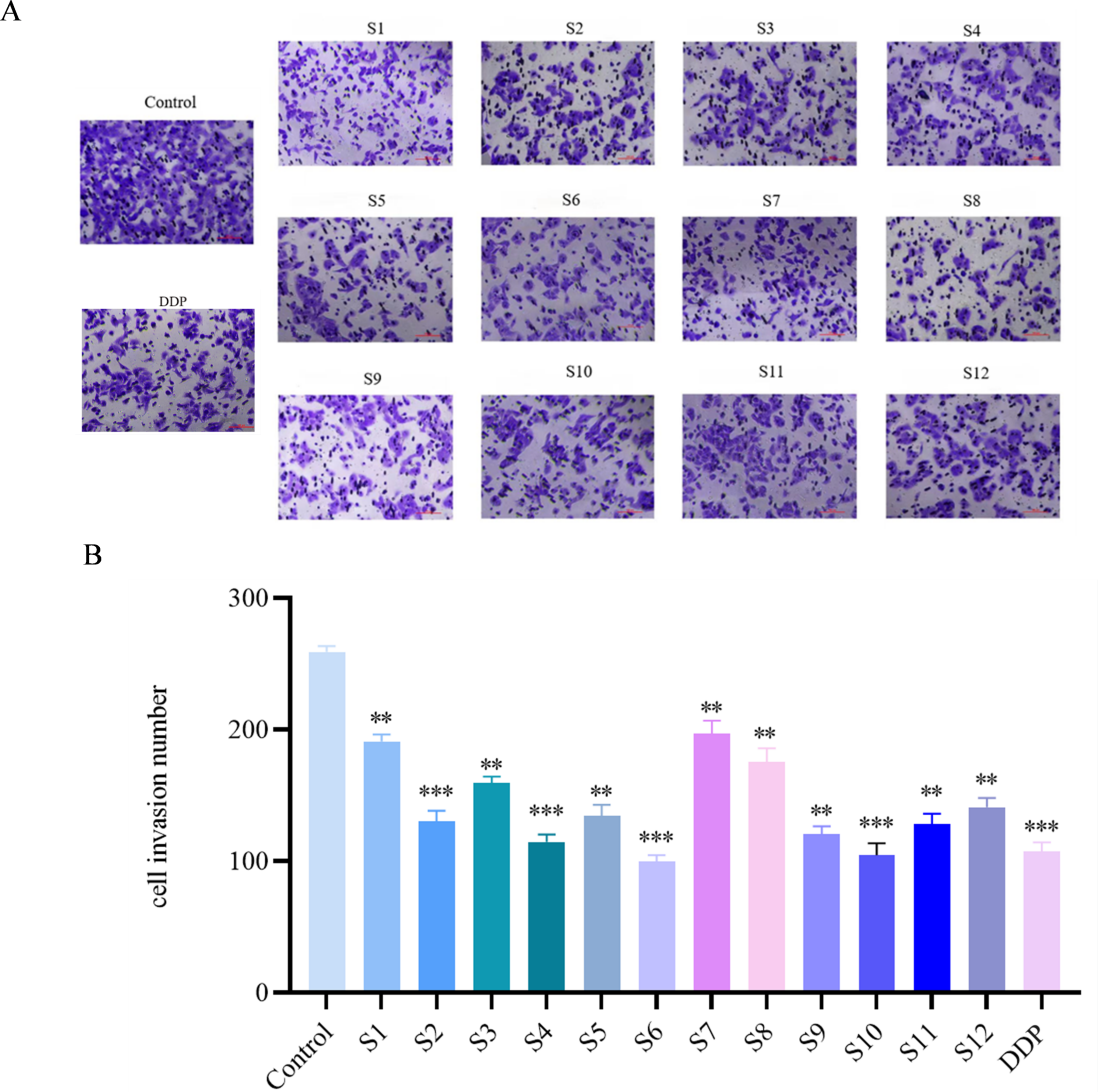
Effect on A549 cell invasion rate of *D. nipponica* Makino subsp. *rosthornii* (n=3) **(A)** representative microscopic images of cell invasion. **(B)** quantification of invaded cells. Data are presented as mean ± SD (*P < 0.05, **P < 0.01, ***P < 0.001 vs control group).

Cell migration is a crucial process in tumor invasion and metastasis (24). In line with this concept, the treatment significantly reduced the invasion rate of A549 cells compared to the blank group, as shown in **Figure 5**.

### 3.6 Spectrum-effect relationship analysis

#### 3.6.1 Pearson correlation analysis

**Figure 6:**
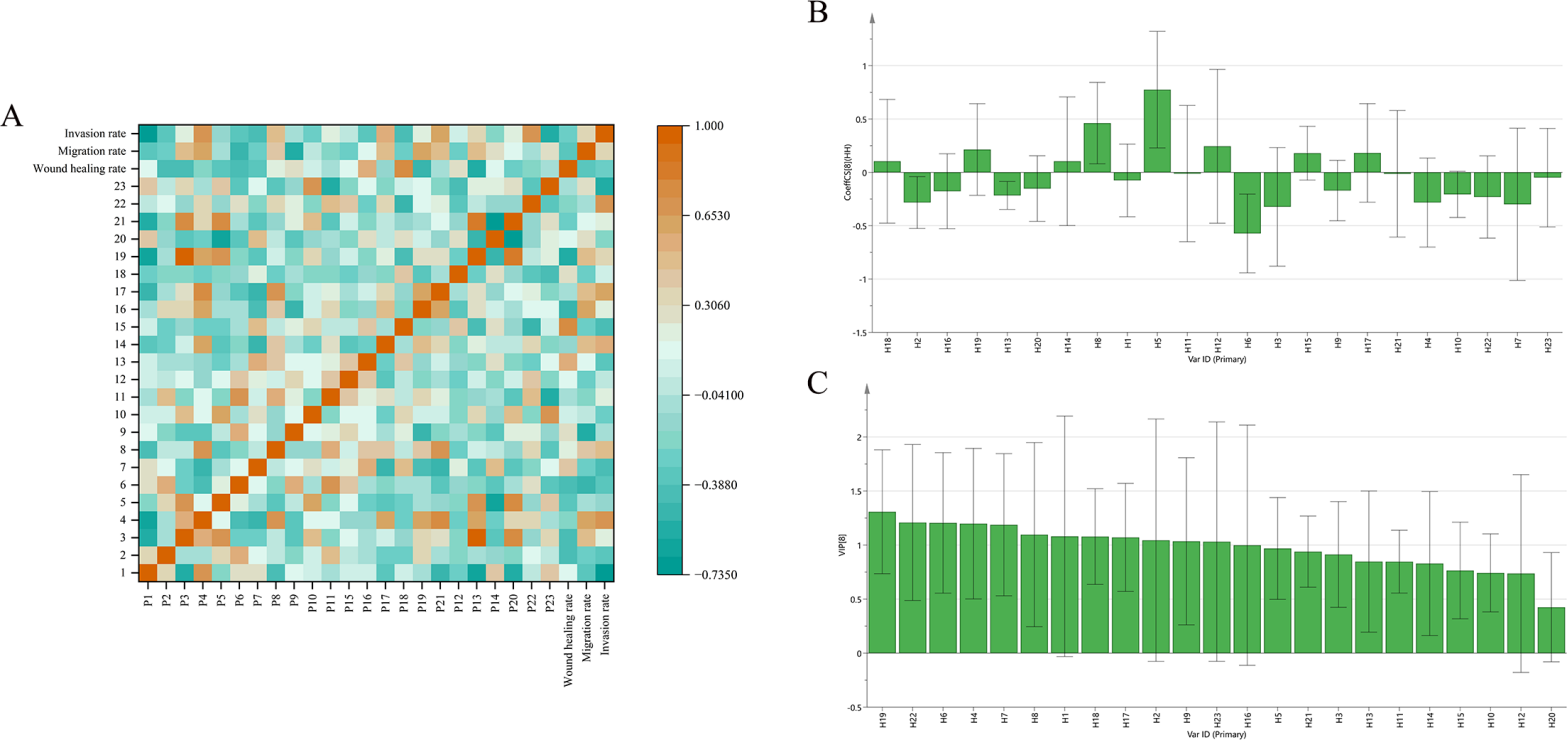
**(A)** Heatmap of pearson correlation analysis between the relative peak areas of 23 common peaks and the cell wound healing rate, migration rate, and invasion rate. Regression coefficients **(B)** and VIP values **(C)** of the peak areas of 23 shared peaks in relation to cell wound healing rate, migration rate, and invasion rate.

SPSS 23.0 software was used to perform Pearson correlation analysis to investigate the relationships between the relative peak areas of 23 common peaks and the cell wound healing rate, migration rate, and invasion rate. The Pearson correlation coefficients were calculated, and the results are shown in **Figure 6A**. Among them, components showing negative correlations indicate antitumor effects, and the greater the absolute value of the correlation coefficient, the stronger the activity. The top five peaks with the strongest negative correlations with the cell wound healing rate were P13 (-0.562), P6 (-0.427), P19 (-0.489), P2 (-0.41), and P7 (-0.386). The top five peaks with the strongest negative correlations with the cell migration rate were P9 (-0.567), P6 (-0.501), P1 (-0.349), P2 (-0.264), and P7 (-0.259). The top five peaks with the strongest negative correlations with the cell invasion rate were P1 (-0.666), P23 (-0.562), P7 (-0.445), P6 (-0.372), and P2 (-0.365).

#### 3.6.2 Partial least squares regression analysis

In the regression model, the Variable Importance in Projection (VIP) score quantifies the contribution of each peak to the antitumor efficacy. A VIP value > 1 was set as the significance threshold. Concurrently, the sign (positive or negative) of the regression coefficient indicates the direction of the relationship between peak area and bioactivity. As shown in **Figure 6B, C**, a high VIP score coupled with a negative regression coefficient signifies a strong inhibitory effect. The top five peaks ranked by VIP score were P22, P6, P4, P7, and P2.

The two analytical methods, Pearson correlation analysis and PLSR analysis, were combined to evaluate the association between the compounds and antitumor activity in *D. nipponica* Makino subsp. *rosthornii*, and to identify potential antitumor active components. Finally, P2, P6 and P7 were screened as potential antitumor active components in *D. nipponica* Makino subsp. *rosthornii*, and were identified as protodioscin, diosgenin and gracillin by UPLC-QTOF-MS analysis, respectively.

### 3.7 Validation of antitumor activity

#### 3.7.1 Inhibition of A549 cell proliferation by three active compounds

**Figure 7:**
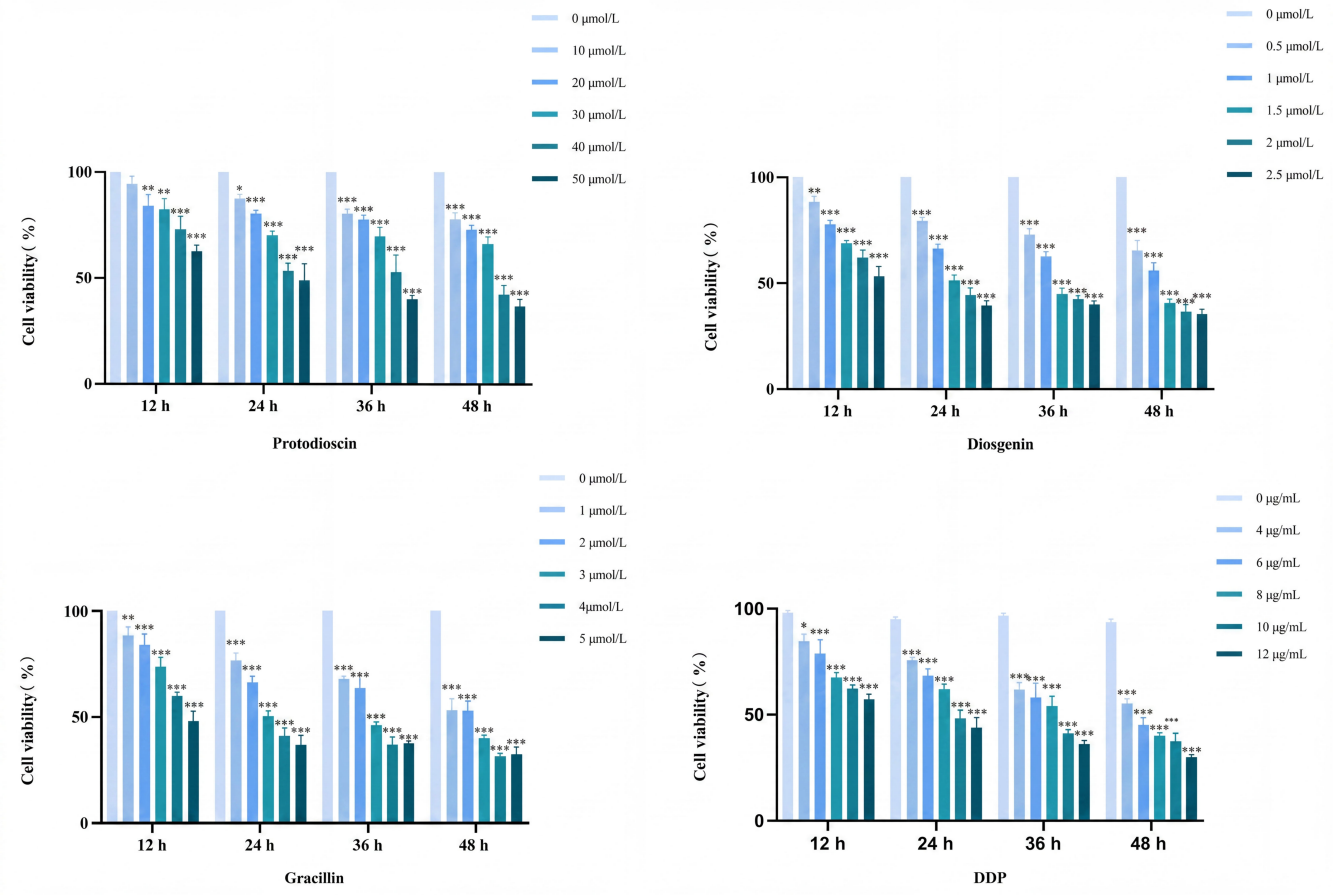
A549 cell proliferation inhibitory effect of potential antitumor factors (n=3) (*P < 0.05, **P < 0.01, ***P < 0.001 vs control group).

A549 cells were seeded in 96-well plates at a density of 7×10³ cells per well and incubated for 24 h. The cells were then divided into several groups: a blank control group, a positive control (cisplatin, DDP), and treatment groups. Preliminary experiments were conducted to determine the effective inhibitory concentration ranges of each compound against A549 cell viability, and the concentration gradients for formal experiments were established accordingly. For the positive control group, cisplatin was added at concentrations of 0, 4, 6, 8, 10, and 12 μg/mL. The treatment groups were exposed to 100 μL of culture medium containing different concentrations of the compounds: diosgenin at concentrations of 0.5, 1, 1.5, 2, and 2.5 μmol/L; protodioscin at concentrations of 10, 20, 30, 40, and 50 μmol/L; and gracillin at concentrations of 1, 2, 3, 4, and 5 μmol/L. All cells were cultured for 24 h. Subsequently, 10 µL of CCK-8 solution was added to each well, followed by incubation for 1 h. The absorbance at 450 nm was measured using a microplate reader to calculate cell viability. As shown in **Figure 7**, all three compounds significantly inhibited the proliferation of A549 cells compared to the blank control group. After 24 h of treatment, the half maximal inhibitory concentrations (IC_50_) of protodioscin, diosgenin, and gracillin against A549 cells were approximately 40, 1.5, and 3 μmol/L, respectively. Therefore, the above concentrations were selected for subsequent experiments.

#### 3.7.2 Potential of three compounds to affect wound healing in A549 cells

**Figure 8:**
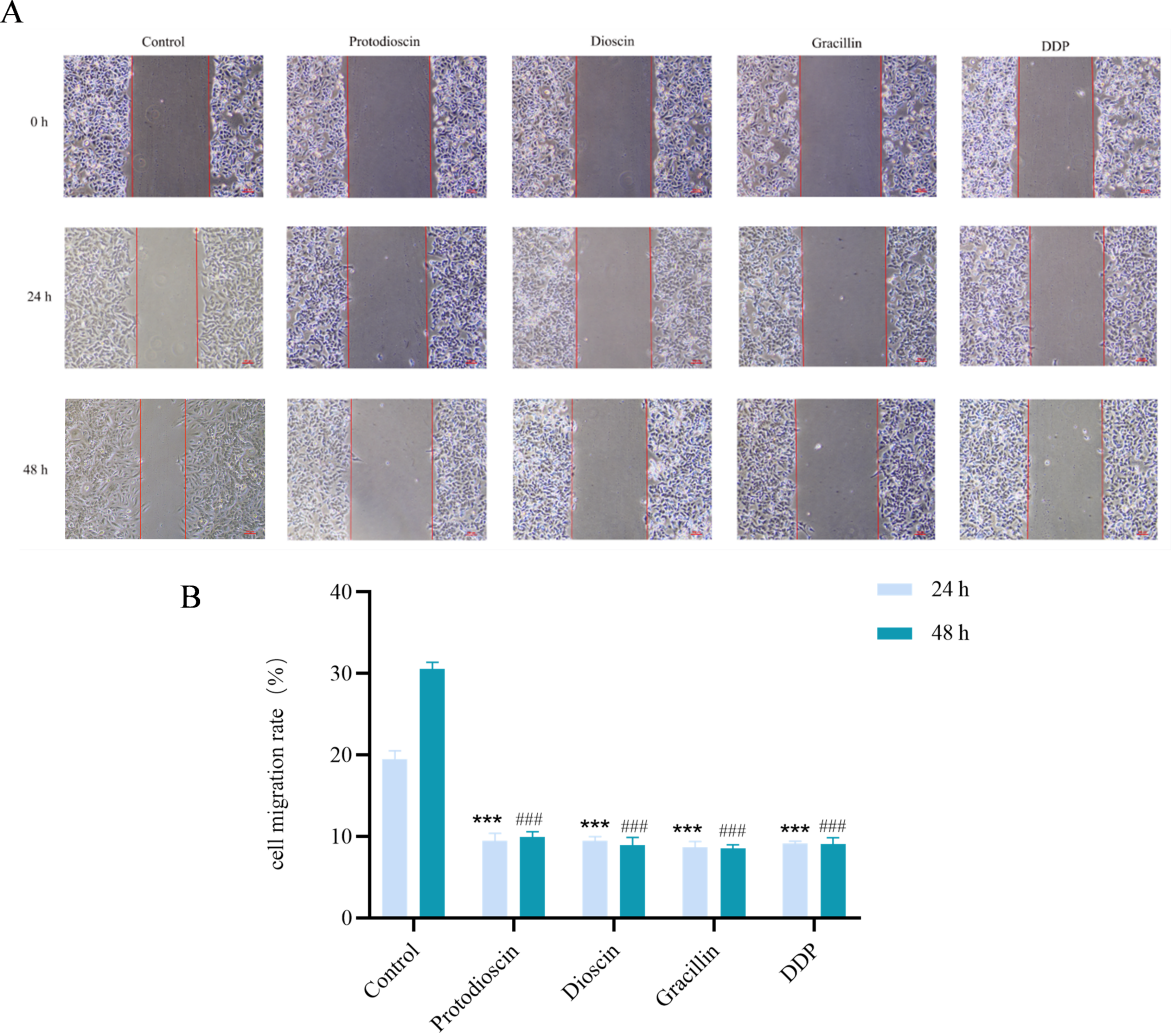
Effect of three compounds on wound closure in A549 cells (n=3). **(A)** representative images showing wound healing at 0, 24, and 48 h. **(B)** quantitative analysis of wound closure rate. Data are presented as mean ± SD (*P < 0.05, **P < 0.01, ***P < 0.001 vs. 24 h control group; ^#^*P* < 0.05, ^##^*P* < 0.01, ^###^*P* < 0.001 vs. 48 h control group).

As shown in **Figure 8**, wound closure was significantly slower in the treatment groups than in the control group after 24 and 48 h of treatment.

#### 3.7.3 Potential of three compounds on the migration of A549 cells

**Figure 9:**
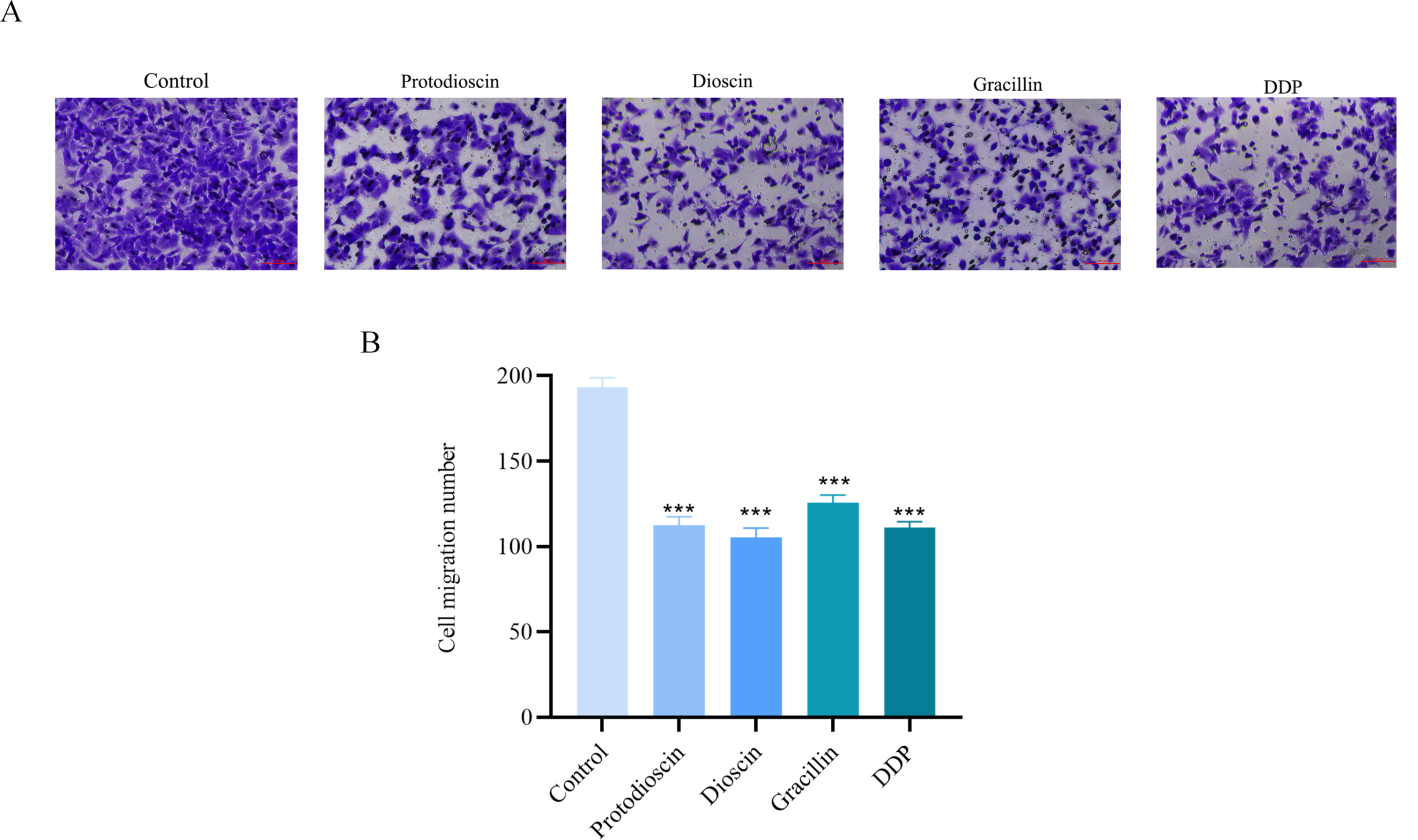
Effect of three compounds on the migration of A549 cells (n=3). **(A)** representative microscopic images of cell migration. **(B)** quantitative analysis of the migration rate. Data are presented as mean ± SD (*P < 0.05, **P < 0.01, ***P < 0.001 vs. control group).

As shown in **Figure 9**, the migration of A549 cells was significantly suppressed in the treatment group compared to the control group.

#### 3.7.4 Potential of three compounds ginger against invasion of A549 cells

**Figure 10:**
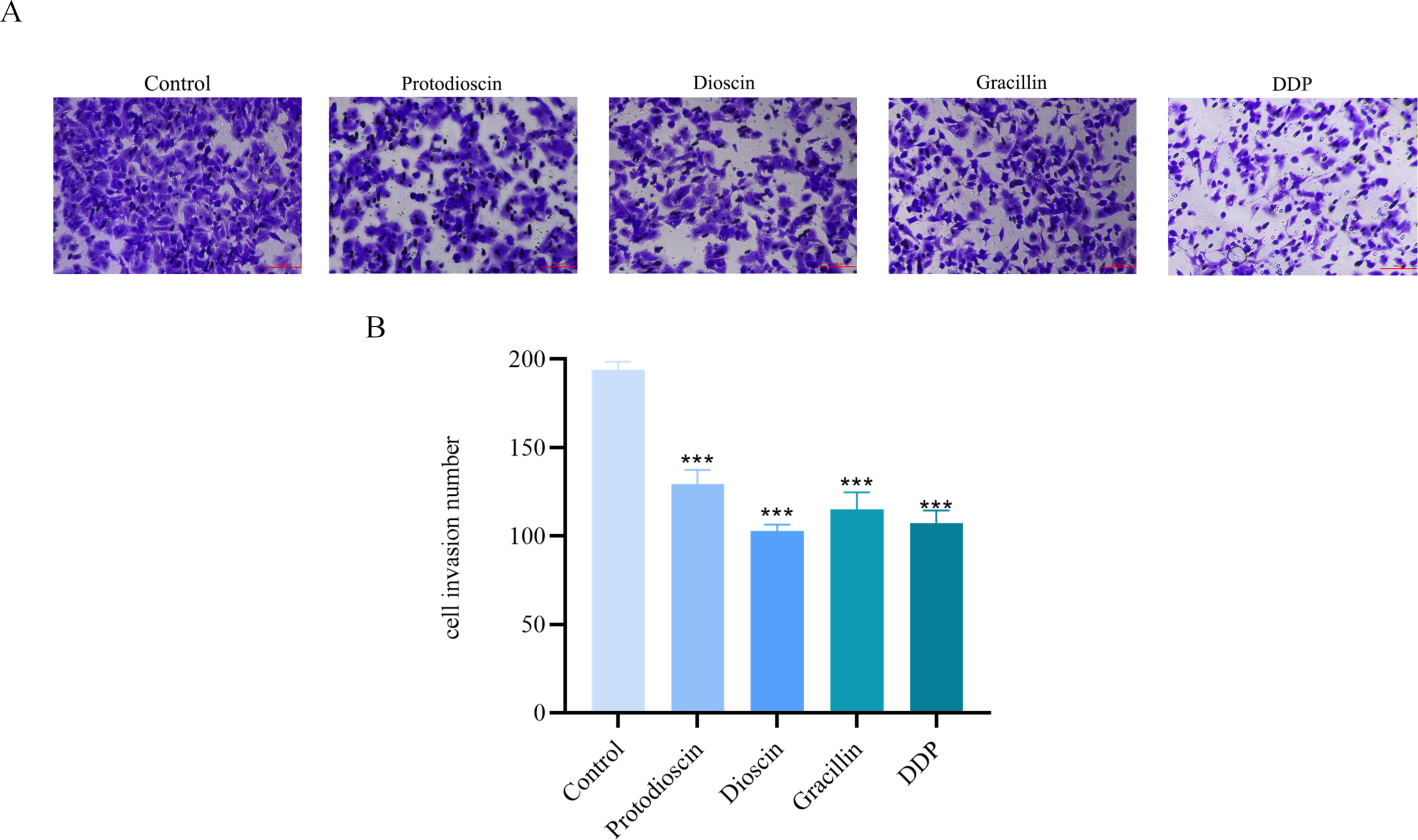
Effect of three compounds on the invasion of A549 cells (n=3). **(A)** representative microscopic images of invaded cells. **(B)** quantitative analysis of cell invasion. Data are presented as mean ± SD (*P < 0.05, **P < 0.01, ***P < 0.001 vs. control group).

As shown in **Figure 10**, the invasion of A549 cells was significantly suppressed in the treatment group compared to the control group.

### 3.8 Exploration of the antitumor mechanism of *D. nipponica* Makino subsp. *rosthornii*

#### 3.8.1 Regulatory effects of potential antitumor active components on protein expression in EMT and oxidative stress pathways

**Figure 11:**
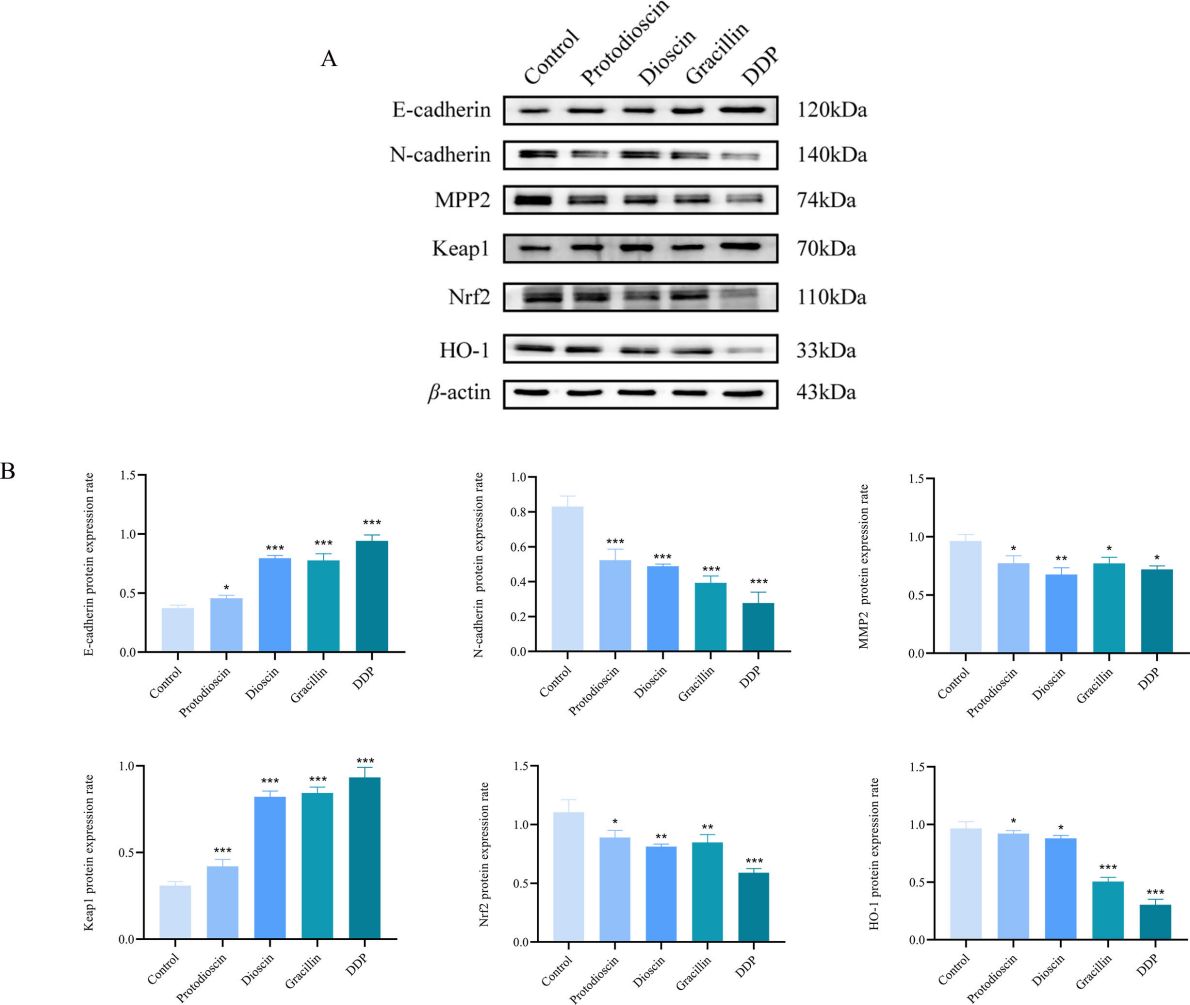
Effects of three compounds on the expression of signaling pathway proteins in A549 cells (n=3) **(A)** representative western blot images of E-cadherin, N-cadherin, MMP2, Keap1, Nrf2, and HO-1. **(B)** quantitative analysis of protein expression levels. Data are presented as mean ± SD (*P < 0.05, **P < 0.01, ***P < 0.001 vs. control group).

As shown in **Figure 11**, the protein expression of N-cadherin and MMP2 was significantly reduced, while that of E-cadherin was increased in the treatment group compared with the control group. These findings suggested that *D. nipponica* Makino subsp. *rosthornii* inhibits the migration and proliferation of A549 cells by down-regulating N-cadherin and MMP2 and up-regulating E-cadherin.

Compared with the blank control group, the expression levels of key proteins in the Keap1/Nrf2/HO-1 oxidative stress pathway—Nrf2 and HO-1—were significantly decreased in A549 cells across all treatment groups, while the expression level of Keap1, a negative regulator of Nrf2, was significantly increased. The Western blot results suggested that *D. nipponica* Makino subsp. *rosthornii* may inhibit the migration and proliferation of A549 cells by modulating the Keap1/Nrf2/HO-1 signaling pathway.

#### 3.8.2 Quantitative real-time PCR

**Figure 12:**
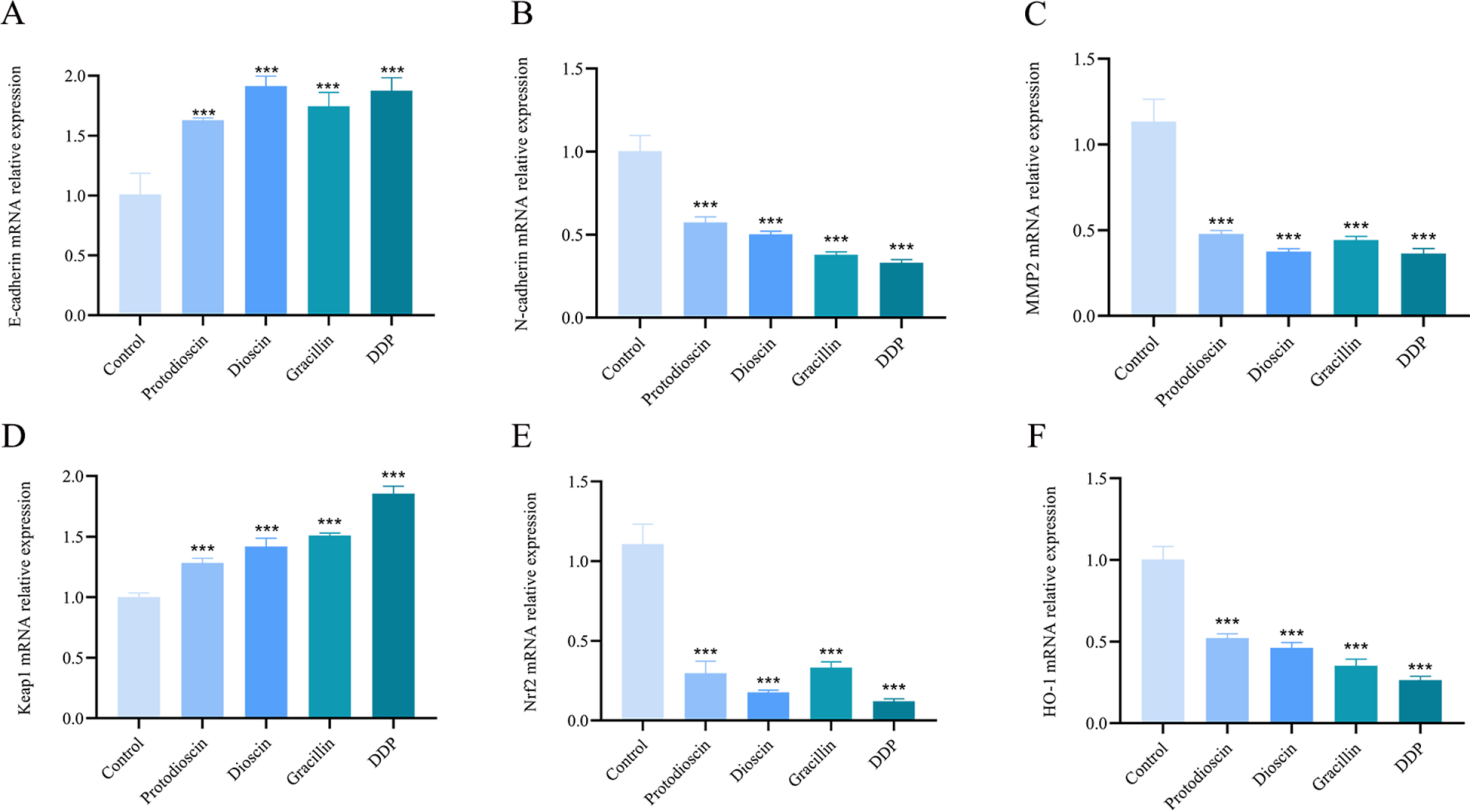
Effects of three compounds on the expression levels of related genes in A549 cells (n=3) [**(A)**: E-cadherin gene; **(B)**: N-cadherin gene; **(C)**: MMP2 gene; **(D)**: Keap1 gene; **(E)**: Nrf2 gene; **(F)**: HO-1 gene) (*P < 0.05, **P < 0.01, ***P < 0.001 vs. Control group).

As shown in **Figure 12**, compared with the blank control group, the mRNA expression levels of N-cadherin and MMP2 were significantly decreased, while the mRNA expression level of E-cadherin was significantly increased in A549 cells across all treatment groups. The qRT-PCR results indicate that the trends of mRNA level changes in the aforementioned genes are consistent with their protein expression patterns, suggesting that *D. nipponica* Makino subsp. *rosthornii* modulates the related targets at both transcriptional and translational levels.

Compared with the blank control group, the mRNA expression levels of Nrf2 and HO-1 were significantly decreased, while the mRNA expression level of Keap1 was significantly increased in A549 cells across all treatment groups. The qRT-PCR results demonstrate that the trends of mRNA level changes in the aforementioned genes are consistent with their protein expression patterns, suggesting that *D. nipponica* Makino subsp. *rosthornii* synchronously regulates the related targets at both transcriptional and translational levels.

## 4 Discussion

The proliferation, migration, and invasion of tumor cells are pivotal steps in tumor progression, and their suppression represents a key strategy in anticancer therapy (25). The scratch assay, which mimics wound healing (26), is used to evaluate two-dimensional cell migration, whereas the Transwell assay employs porous membranes to simulate the extracellular matrix barrier, enabling the assessment of cell migration (in the absence of Matrigel) and invasion (in the presence of Matrigel), thereby offering a closer approximation of the *in vivo* microenvironment (27). Spectrum-effect relationship analysis establishes an association model between chemical constituents and pharmacological efficacy in traditional Chinese medicine, enabling the screening of key components that significantly contribute to the bioactivity (28). Using UPLC-QTOF/MS, a systematic chemical analysis of 12 batches of *D. nipponica* Makino subsp. *rosthornii* from different origins was conducted, leading to the identification of 23 chemical constituents. Subsequently, multivariate statistical methods, including Pearson correlation analysis and partial least squares regression (PLSR), were applied to elucidate the quantitative relationship between chemical composition and antitumor activity at the molecular level. Through Pearson correlation analysis and PLSR, protodioscin, diosgenin, and gracillin were identified as key efficacy-contributing components (VIP > 1, *P* < 0.05). This finding aligns with previous reports on the significant antitumor activity of steroidal saponins in the genus *Dioscorea* (29).

The CCK-8 assay showed that the three compounds protodioscin, diosgenin and gracillin significantly inhibited the proliferation of A549 cells with IC_50_ values consistent with previous reports (30, 31), and all three compounds effectively suppressed the migration and invasion abilities of A549 cells. This phenomenon may be related to the inhibition of epithelial−mesenchymal transition (EMT) (29). Previous studies have shown that various natural products, including wogonin, curcumin, and Sophora flavescens, inhibit EMT in lung cancer cells by regulating the expression of E−cadherin and N−cadherin (32–34). Therefore, the saponins investigated in this study may exert their effects through a similar mechanism.

Most cancer-related deaths are caused by the proliferation and metastasis of primary tumor cells (35). Therefore, identifying key molecules that regulate cancer cell proliferation and metastasis and elucidating their molecular mechanisms may help identify potential therapeutic targets and could eventually inform strategies aimed at improving treatment outcomes, although further *in vivo* and clinical studies are required. Matrix metalloproteinases (MMPs) play a central role in tumor invasion and metastasis by degrading the extracellular matrix (ECM) (36). In particular, MMP-2 cleaves type IV collagen networks in the ECM, removing physical constraints on tumor cell movement and thereby promoting an invasive phenotype. Inhibition of MMP-2 gene expression reduces the migration and invasion abilities of tumor cells (37). The expression level of E-cadherin is inversely correlated with tumor malignancy. Loss of E-cadherin weakens intercellular connections and enhances the ability of tumor cells to detach from the primary site, whereas restoration of its expression effectively blocks metastatic progression. In contrast, overexpression of N-cadherin promotes both basement membrane penetration and specific tumor cell–endothelial cell adhesion. Therefore, targeted inhibition of N-cadherin has become an important strategy in anti-metastatic therapy (38–40).

In the regulation of oxidative stress, heme oxygenase−1 (HO−1) catalyzes the breakdown of heme to generate CO and biliverdin, thereby enhancing the survival advantage of tumor cells in hostile microenvironments (41). Nuclear factor erythroid 2−related factor 2 (Nrf2) is a central regulator of redox homeostasis. Under physiological conditions, its expression is maintained at low levels through Keap1−dependent ubiquitination and degradation, but it is frequently dysregulated in tumors. Aberrantly activated Nrf2 translocate into the nucleus and upregulates antioxidant genes such as HO−1, NQO1, and glutathione−synthesizing enzymes, protecting tumor cells from ROS damage while promoting proliferation, angiogenesis, and therapy resistance, ultimately leading to poor prognosis. Enhancing Keap1 expression or suppressing Nrf2 signaling at the genetic level can restore ROS homeostasis and inhibit tumor progression (42, 43).

This study confirmed the antitumor potential of *D. nipponica* Makino subsp. *rosthornii* through cell proliferation, migration, wound healing, and invasion assays. In future work, we will further analyze its effects on cell cycle and apoptosis to complete the experimental system. On this basis, future research can further investigate the *in vivo* antitumor effects of *D. nipponica* Makino subsp. *rosthornii* and its active components. By establishing tumor animal models, the *in vivo* antitumor efficacy and safety of the extracts of *D. nipponica* Makino subsp. *rosthornii* (including protodioscin, diosgenin, and gracillin) can be validated, clarifying their antitumor effects in the context of complex metabolic and immunoregulatory processes *in vivo*. Meanwhile, in addition to A549 cells, the study can be extended to other tumor cell lines and more cancer types to assess the generalizability of its antitumor effects. Regarding the regulatory mechanism of the Keap1/Nrf2/HO-1 pathway, further in−depth exploration can be conducted using pathway−specific inhibitors or gene silencing approaches, which will help to more comprehensively elucidate the antitumor potential of *D. nipponica* Makino subsp. *rosthornii* and provide a scientific basis for subsequent *in vivo* studies.

## 5 Conclusion

This study identified antitumor compounds from *D. nipponica* Makino subsp. *rosthornii* and investigated their underlying mechanisms of action. The ethanol extract of *D. nipponica* subsp. *rosthornii* significantly suppressed the proliferation, migration, and invasion of A549 cells, as demonstrated by CCK-8, wound healing, and Transwell migration and invasion assays. Based on spectrum-effect relationship analysis, protodioscin, diosgenin, and gracillin were screened as potential antitumor constituents in *D. nipponica* Makino subsp. *rosthornii*.

For chemical composition analysis, the study used UPLC-QTOF/MS technology, which ultimately led to the identification of 23 compounds. The relationship between these chemical constituents and their antitumor activity was investigated using spectrum effect-relationship analysis. The results showed that protodioscin, diosgenin, and gracillin. were compounds with potential antitumor activity. And the potential antitumor factors screened for them were validated. Proliferation and metastasis are crucial processes in tumor cells. It was found that protodioscin, diosgenin, and gracillin may exert antitumor effects by suppressing the expression of N-cadherin, MMP2, HO-1, and Nrf2, while promoting the expression of E-cadherin and Keap1.

This study not only developed comprehensive methods, such as UPLC-QTOF-MS mapping and spectrum-effect relationship analysis, but also offered a detailed analysis of the bioactive constituents of the Chinese herbal medicine, along with experimental evidence supporting their antitumor mechanisms.

## Data Availability

The original contributions presented in the study are included in the article/**Supplementary Material**. Further inquiries can be directed to the corresponding authors.
