# Supplementary material for: Screening, validation, and mechanism study of antitumor components from Dioscorea nipponica Makino subsp. rosthornii based on spectrum-effect relationship analysis
Source: Front Oncol. 2026 Apr 29;16:1836431. doi: 10.3389/fonc.2026.1836431 (PMC13167525; doi:10.3389/fonc.2026.1836431)
Supplement: Supplementary file 1 [file Table1.docx]

Supplementary Material

# Supplementary Table

Table S1 Inhibitory effect of *Dioscorea nipponica* Makino subsp. *rosthornii* on A549 cell proliferation: mean, standard deviation, and 95% confidence interval (*n* = 3).

| Sample ID | Concentration (μg/mL) | Mean | SD | 95% CI |
| --- | --- | --- | --- | --- |
| S1 | 250 μg/mL | 79.25 | 11.76 | (60.54-97.96) |
|  | 300 μg/mL | 75.08 | 12.33 | (55.46-94.70) |
|  | 350 μg/mL | 67.75 | 19.29 | (37.06-98.44) |
|  | 400 μg/mL | 60.92 | 15.64 | (36.03-85.80) |
|  | 450 μg/mL | 44.25 | 15.65 | (19.353-19.35) |
| S2 | 250 μg/mL | 90.08 | 7.927 | (77.47-102.7) |
|  | 300 μg/mL | 81.25 | 10.63 | (64.34-98.16) |
|  | 350 μg/mL | 71.08 | 10.16 | (54.92-87.24) |
|  | 400 μg/mL | 53.25 | 16.88 | (26.39-80.11) |
|  | 450 μg/mL | 41.17 | 18.42 | (11.86-70.48) |
| S3 | 250 μg/mL | 87.58 | 7.753 | (75.25-99.92) |
|  | 300 μg/mL | 83.58 | 9.886 | (67.85-99.31) |
|  | 350 μg/mL | 73.17 | 14.02 | (50.85-95.48) |
|  | 400 μg/mL | 56.25 | 21.55 | (21.95-90.55) |
|  | 450 μg/mL | 50.25 | 21.87 | (15.45-85.05) |
| S4 | 250 μg/mL | 85.25 | 8.85 | (71.17-99.33) |
|  | 300 μg/mL | 78.25 | 9.159 | (63.68-92.82) |
|  | 350 μg/mL | 62.92 | 12.45 | (43.11-82.72) |
|  | 400 μg/mL | 49.92 | 10.96 | (32.48-67.35) |
|  | 450 μg/mL | 44.83 | 12.66 | (24.68-64.98) |
| S5 | 250 μg/mL | 87.25 | 10.22 | (70.98-103.50) |
|  | 300 μg/mL | 83.5 | 11.93 | (64.52-102.50) |
|  | 350 μg/mL | 76.5 | 19.83 | (44.94-108.1) |
|  | 400 μg/mL | 54.5 | 14.56 | (31.33-77.67) |
|  | 450 μg/mL | 42.42 | 15.73 | (17.39-67.44) |
| S6 | 250 μg/mL | 85.08 | 9.422 | (70.09-100.1) |
|  | 300 μg/mL | 80.08 | 9.597 | (64.81-95.35) |
|  | 350 μg/mL | 70.92 | 12.2 | (51.51-90.32) |
|  | 400 μg/mL | 57.33 | 16.88 | (30.48-84.19) |
|  | 450 μg/mL | 47.5 | 17.25 | (20.05-74.95) |
| S7 | 250 μg/mL | 88.08 | 8.034 | (75.30-100.9) |
|  | 300 μg/mL | 78.5 | 9.485 | (63.41-93.59) |
|  | 350 μg/mL | 64.67 | 10.81 | (47.46-81.87) |
|  | 400 μg/mL | 49.33 | 11.8 | (30.56-68.11) |
|  | 450 μg/mL | 42.5 | 14.19 | (19.92-65.08) |
| S8 | 250 μg/mL | 90.42 | 7.411 | (78.62-102.2) |
|  | 300 μg/mL | 72.67 | 8.823 | (58.63-86.71) |
|  | 350 μg/mL | 63.75 | 8.234 | (50.65-76.85) |
|  | 400 μg/mL | 44.17 | 8.884 | (30.03-58.30) |
|  | 450 μg/mL | 27.42 | 6.356 | (17.30-37.53) |
| S9 | 250 μg/mL | 84.92 | 13.03 | (64.19-105.6) |
|  | 300 μg/mL | 82.33 | 11.79 | (63.57-101.1) |
|  | 350 μg/mL | 75.75 | 11.17 | (57.98-93.52) |
|  | 400 μg/mL | 70.83 | 10.65 | (53.88-87.79) |
|  | 450 μg/mL | 44.5 | 21.17 | (10.81-78.19) |
| S10 | 250 μg/mL | 68.17 | 20.81 | (35.05-101.3) |
|  | 300 μg/mL | 58 | 22.89 | (21.58-94.42) |
|  | 350 μg/mL | 50.83 | 18.87 | (20.80-80.86) |
|  | 400 μg/mL | 43.83 | 11.5 | (25.54-62.13) |
|  | 450 μg/mL | 34 | 10.24 | (17.71-50.29) |
| S11 | 250 μg/mL | 75.92 | 16.21 | (50.13-101.7) |
|  | 300 μg/mL | 72.33 | 11.8 | (53.55-91.12) |
|  | 350 μg/mL | 62.25 | 18.92 | (32.15-92.35) |
|  | 400 μg/mL | 41.92 | 14.4 | (19.00-64.83) |
|  | 450 μg/mL | 29.17 | 8.167 | (16.17-42.16) |
| S12 | 250 μg/mL | 79.75 | 10.14 | (63.61-95.89) |
|  | 300 μg/mL | 75.58 | 10.21 | (59.33-91.84) |
|  | 350 μg/mL | 67.67 | 9.68 | (52.26-83.07) |
|  | 400 μg/mL | 52.42 | 18.36 | (23.20-81.63) |
|  | 450 μg/mL | 36.58 | 20.39 | (4.145-69.02) |
